# Causal effects of gut microbiome on endometriosis: a two-sample mendelian randomization study

**DOI:** 10.1186/s12905-023-02742-0

**Published:** 2023-11-30

**Authors:** Ziyu Liu, Peigen Chen, Liling Luo, Qianru Liu, Hao Shi, Xing Yang

**Affiliations:** 1 Reproductive Medicine Research Center, The Sixth Affiliated Hospital, Sun Yat-sen University, Guangzhou, Guangdong People’s Republic of China; 2 Guangdong Engineering Technology Research Center of Fertility Preservation, Guangzhou, Guangdong China

**Keywords:** Endometriosis, Gut microbiota, Mendelian randomization study, Causal effect

## Abstract

**Background:**

Previous studies have shown observational associations between the gut microbiota and endometriosis; however, the causal nature of such associations remains unclear. This study aimed to analyze the genetic causal relationship between the two.

**Methods:**

A gut microbiome genome-wide association study conducted by the MiBioGen consortium was used as exposure data, and summary statistics of endometriosis were obtained from the FinnGen consortium R8 release data. Inverse variance weighted, MR-Egger, weighted median, weighted model, and simple model analyses were applied to examine the causal relationship, and sensitivity analyses were conducted to validate the robustness of the results.

**Results:**

The results showed that, out of 211 gut microbiome taxa, Clostridiales_vadin_BB60_group, Oxalobacteraceae, Desulfovibrio, Haemophilus, and Holdemania had protective effects on endometriosis, while Porphyromonadaceae and Anaerotruncus might contribute to the development of endometriosis. Heterogeneity and pleiotropy analyses confirmed the robustness of the results.

**Conclusion:**

The two-sample Mendelian randomization analysis conducted in this study identified specific intestinal flora with a causal relationship with endometriosis at the genetic level, offering new insights into the gut microbiota-mediated development mechanism of endometriosis.

**Supplementary Information:**

The online version contains supplementary material available at 10.1186/s12905-023-02742-0.

## Introduction

Endometriosis is a prevalent chronic inflammatory condition with severe consequences on reproductive and general health, characterized by the growth of functional endometrial glands and stroma outside the uterine cavity. The most common ectopic sites include the ovaries, fossa ovarica, and uterosacral ligaments [1]. Symptoms primarily include dysmenorrhea and subfertility, but may also present as non-cyclical or chronic pelvic pain, deep dyspareunia, and dyschezia [2]. Endometriosis affects approximately 10% of reproductive-aged women worldwide, affecting approximately 175 million individuals [3, 4], and its exact etiology remains largely unknown, despite being first described almost a century ago. Current treatment strategies, including pain medication, hormonal therapy, surgical excision of endometriotic lesions, and hysterectomy, have negative side effects and are unable to prevent recurrences [5]. Therefore, exploring the etiology of endometriosis is essential for the development of effective and minimally damaging treatment options.

Gut microbiota is a dynamic and complex community of ecological microbes that inhabit the human intestine, often referred to as the “forgotten organ” [6]. Recent evidence suggests that the gut microbiota is closely linked to host health and is involved in the development of various complex human diseases, including endometriosis [7, 8]. In addition to gynecological symptoms, up to 90% of patients with endometriosis experience gastrointestinal symptoms, including bloating, nausea, constipation, diarrhea, and vomiting [9, 10]. Consequently, it has been postulated that the gut microbiome may plays a pivotal role in the orchestration of endometriosis and related disorders. Several studies have revealed that patients with endometriosis experience gut microbiome dysbiosis and decreased species richness. For example, Svensson et al. confirmed that there was a marked difference in the abundance of 12 bacteria belonging to the classes Bacilli, Bacteroidia, Clostridia, Coriobacteriia, and Gammaproteobacteria between patients with endometriosis and the control group [7]. Ata et al. observed that the gut microbiota composition was altered in patients with endometriosis compared with that in the control group. Specifically, they found that more women in the stage 3/4 endometriosis group presented with a Shigella/Escherichia dominant stool microbiome [11]. Yu et al. also reported a reduction in the diversity of gut microbiota in patients with endometriosis. However, they found that the abundances of Actinobacteria, Cyanobacteria, Saccharibacteria, Fusobacteria, and Acidobacteria was significantly increased [12]. Additionally, fecal metabolomics has demonstrated differences in the gut microbiota and associated metabolites in mice with and without endometriosis [13]. Nonetheless, in observational studies, the relationship between the gut microbiota and endometriosis is susceptible to confounding factors such as age, environment, dietary patterns, and lifestyle, making it difficult to control these factors effectively [14]. Accordingly, these issues limit the establishment of a causal link between the gut microbiota and endometriosis.

Mendelian randomization (MR) is a framework that integrates summary data from genome-wide association studies (GWAS) [15] to evaluate causality from exposure to an outcome. This approach leverages genetic variants as instrumental variables (IVs) [16], taking advantage of the random allocation of genotypes from parents to offspring to estimate associations with outcomes that are not confounded by common factors. Therefore, a plausible causal sequence has been established [17]. Two-sample MR analysis can further combine the single nucleotide polymorphism (SNP)-exposure and SNP-outcome associations from independent GWAS analyses to generate a single causal estimate. With the rapid expansion of GWAS in the fields of gut microbiota and psychiatric disorders, large-scale summary statistics have become increasingly accessible, enabling two-sample MR analysis with greater statistical power [18, 19]. The present study employed gut microbiome taxa as the exposure and endometriosis as the outcome in a two-sample MR analysis to explore causal relationships and provide a theoretical foundation for future investigations of the complex mechanisms underlying endometriosis.

## Materials and methods

### Study design and the assumption of MR

**Fig. 1:**
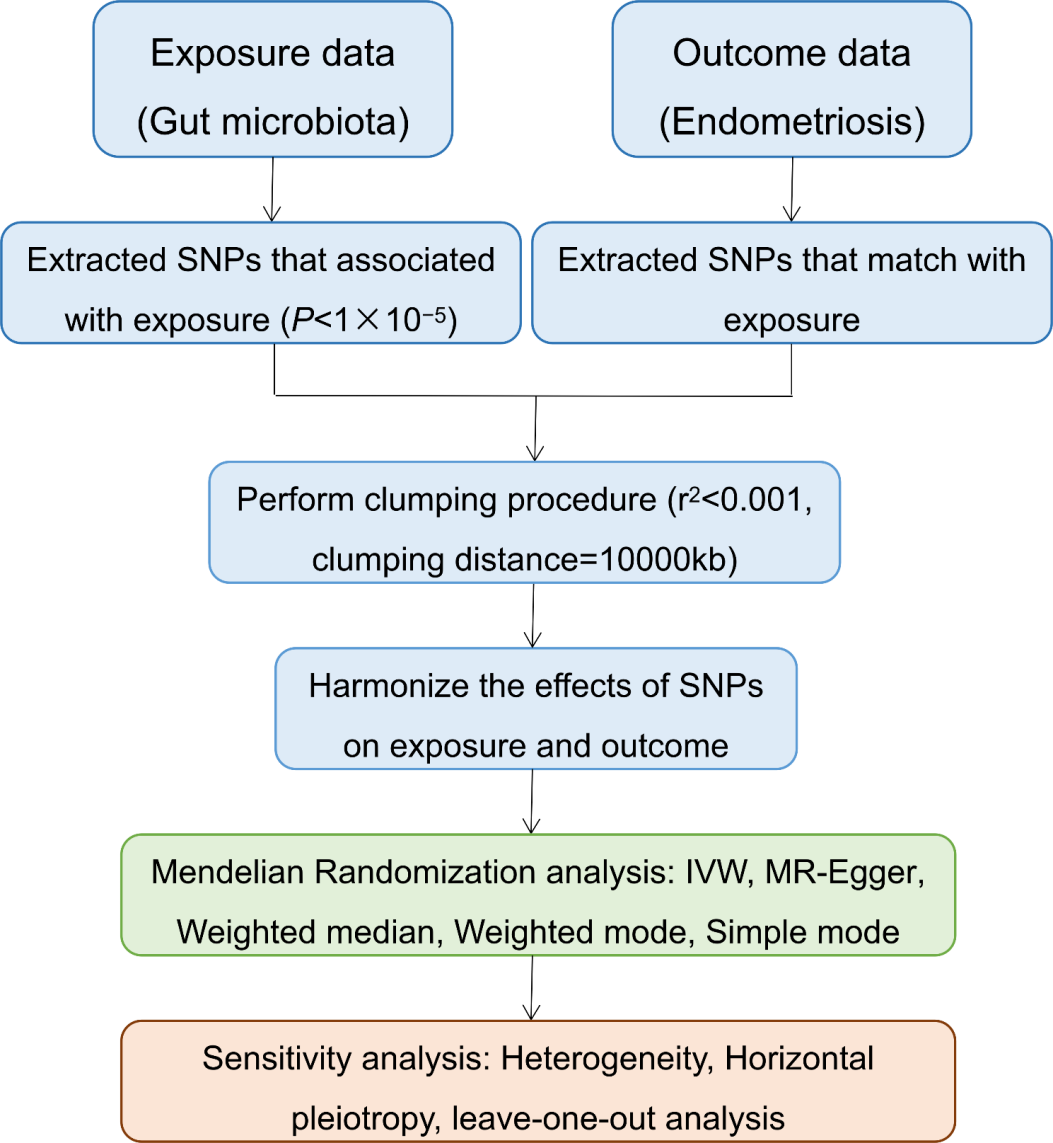
Diagrammatic description of the whole workflow in MR analysis. A flowchart of the whole MR analysis was displayed in this figure. SNP, single nucleotide polymorphism; IVW, inverse variance-weighted

**Fig. 2:**
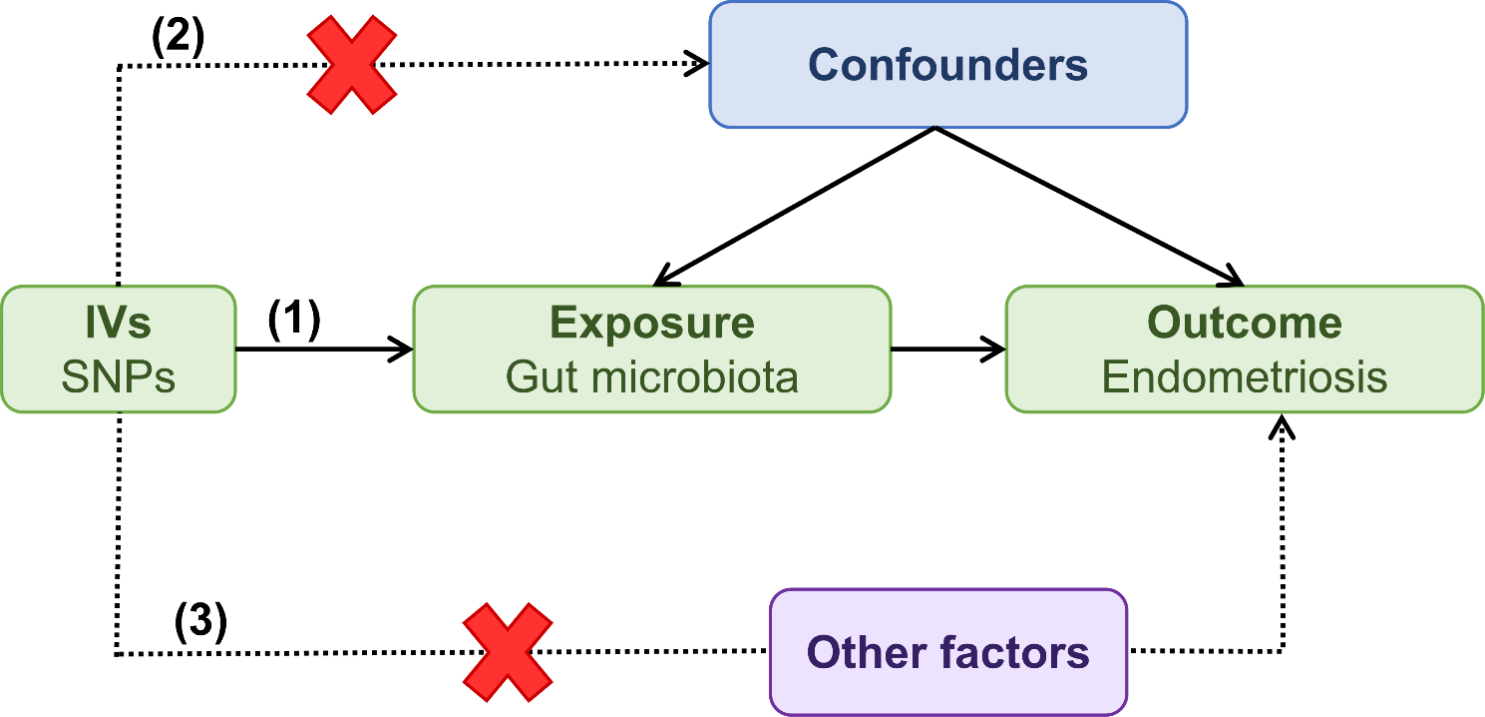
Three main assumptions of Mendelian randomization. (1) IVs selected from datasets were related with exposure; (2) IVs were not related with any confounder factors; (3) IVs can affect outcomes only through exposure, but not in other ways. SNP, single nucleotide polymorphism; IVW, inverse variance-weighted

Figure 1 depicts the flowchart of the study, which shows that the gut microbiota was utilized as the exposure, whereas endometriosis was envisaged as the outcome. Using GWAS summary data for gut microbiota and endometriosis, eligible instrumental variables (IVs) were meticulously screened for MR analysis to discern the causal relationship between the gut microbiota and endometriosis. For optimal outcomes, the two-sample MR was conducted under the following provisions [20]: (1) IVs chosen from datasets were correlated with the exposure; (2) IVs were not related to any confounding factors; and (3) IVs could have an impact on the outcomes merely through exposure and not through other pathways (Fig. 2). All datasets included in this study were publicly accessible, and each GWAS deployed in this study received ethical approval from the respective institutions.

### GWAS summary data for exposure

The present study utilized GWAS summary statistics derived from the MiBioGen, the most comprehensive meta-analysis of the gut microbiome (https://mibiogen.gcc.rug.nl/) [18]. The cohort comprised 18,340 individuals from 24 diverse populations, encompassing countries including the USA, Canada, Israel, South Korea, Germany, Denmark, the Netherlands, Belgium, Sweden, Finland, and the UK. Microbial taxonomy was obtained by sequencing of the variable regions V4, V3-V4, and V1-V2 of the 16 S rRNA gene, followed by direct taxonomic binning analysis [18]. The taxonomic classification included 211 taxa (encompassing 131 genera, 35 families, 20 orders, 16 classes, and 9 phyla) from 122,110 variant sites analyzed across diverse populations, with unknown taxa being excluded from the study [18].

### GWAS summary data for outcome

The summary statistics of endometriosis GWAS were obtained from the FinnGen research project, which integrates genetic data of disease endpoints from the Finnish Biobank and the Finnish Health Registry (https://r8.finngen.fi/). The sample size included 13,456 endometriosis cases and 100,663 controls, of European ancestry. Detailed information regarding the participating cohorts, genotypes utilized, endpoint definitions, and association tests performed by the FinnGen consortium can be found on the official webpage (https://finngen.gitbook.io/documentation/).

### IVs selection

To ensure the accuracy of the conclusions regarding the causal effect of enteric microbiota on endometriosis, quality control steps were undertaken in the selection of genetic predictors associated with microbiome features. The following criteria were employed for the selection of IVs: (1) SNPs associated with gut microbiota taxa that reached the genome-wide significance threshold (*P* < 5 × 10^− 8^). Because of the limited availability of eligible IVs (*P* < 5 × 10^− 8^), a more comprehensive threshold *(P* < 1 × 10^− 5^) was employed to obtain a more inclusive result [21]; (2) LD analysis was performed (R^2^ < 0.001, clumping distance = 10,000 kb) based on the European-based 1,000 Genome Projects, and SNPs that did not meet the requirements were excluded; and (3) when palindromic SNPs were present, the forward strand alleles were inferred using allele frequency information.

To assess the strength of the selected SNPs, the F-statistics for each bacterial taxon were calculated using the following equation: F = R^2^(N − K − 1)/K(1 − R^2^), where R^2^ is the proportion of exposure variance explained by the IVs, n is the sample size, and k is the number of IVs. An F-statistic > 10 indicate the absence of significant weak instrumental bias [22].

### MR analysis

The present study utilized five commonly used MR methods, namely the inverse variance weighted (IVW), MR-Egger, weighted median, simple mode, and weighted mode, to investigate the causal relationship between the human gut microbiome composition and endometriosis risk. IVW was employed as the primary approach for calculating causal effect values to achieve unbiased estimates, with the other four methods utilized as supplements. IVW determines the causal effect of exposure on the outcome by aggregating the ratio estimates for each SNP, equivalent to a weighted regression of SNP-outcome effects against SNP-exposure effects [23]. MR-Egger is a technique that provides a causal effect through the slope coefficient of Egger regression, and also detects minor study bias, yet it may reduce statistical power [20]. The weighted median approach yields unbiased estimates, even in the presence of up to 50% invalid instrumental variables [24]. The simple mode is a model-based evaluation that confers pleiotropic robustness [25], whereas weighted mode is highly sensitive to hard throughput collection [26].

### Sensitivity analysis

Sensitivity analyses were conducted to assess the compatibility of instrumental variables, by employing heterogeneity measures. Cochran’s Q (IVW) and Rucker’s Q (MR-Egger) statistics were utilized to detect heterogeneity in the MR analysis, where *P* < 0.05 was considered indicative of heterogeneity in the instrumental variables [27]. Additionally, the intercept of the MR-Egger regression test provides an estimate of horizontal pleiotropy, indicating that the instrumental variables are associated with outcomes through mechanisms other than direct causality. Horizontal pleiotropy may have resulted in a false-positive associations (*P* < 0.05). Moreover, to investigate whether the causal signal was driven by a single nucleotide polymorphism [28], a leave-one-out analysis was performed. Finally, Mendelian randomization pleiotropy residual sum and outlier (MR-PRESSO) analysis was implemented as a distortion test to detect potential outliers in MR analysis.

### Statistical analysis

All statistical analyses, including MR and sensitivity analyses, were conducted using R software (version 4.2.3). The TwoSampleMR [25] and MR-PRESSO packages [29] were employed for the MR analyses.

## Results

### Selection of IVs

Initially, we detected 214, 460, 1,617, 263, and 115 SNPs associated with the composition of the gut microbiota at the class, family, genus, order, and phylum levels, respectively, at the suggested significance threshold of *P* < 1 × 10^− 5^ (Table S1). After implementing a comprehensive quality control process, we identified 15 SNPs that were significantly associated with the Clostridiales_vadin_BB60_group, 14 SNPs associated with Oxalobacteraceae, 9 SNPs associated with Porphyromonadaceae, 13 SNPs associated with Anaerotruncus, 10 SNPs associated with Desulfovibrio, 9 SNPs associated with Haemophilus, and 14 SNPs associated with Holdemania (Table S2). Remarkably, the F-statistics for each instrumental variable demonstrating a noteworthy correlation with the gut microbiome surpassed 10, indicating negligible evidence of weak instrument bias.

### Causal effects of gut microbiota on endometriosis

**Fig. 3:**
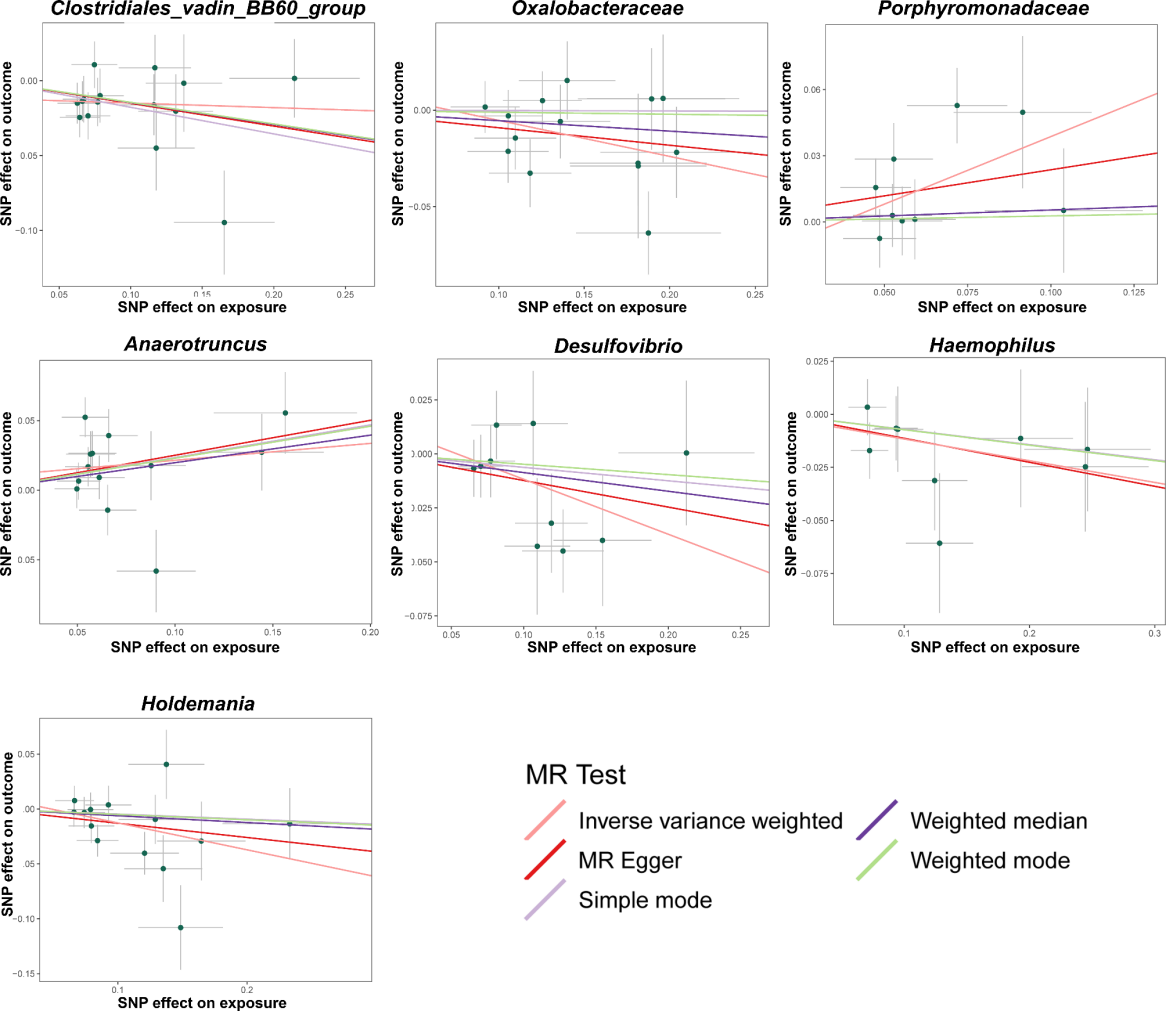
Scatter plots for the causal association between gut microbiota and endometriosis. The effect of the gut microbiome on endometriosis is calculated through single nucleotide polymorphisms (SNPs), which provide an association between the gut microbiome and endometriosis through five Mendelian randomization methods. The x-axis values represent the effect of SNPs on the gut microbiome. The y-axis values represent the effect of the SNPs on endometriosis. MR, Mendelian randomization; SNP, single nucleotide polymorphism

Five MR methods were used to test the causal association between each bacterial component and the endometriosis. As presented in Table 1; Fig. 3, seven bacteria, namely Clostridiales_vadin_BB60_group, Oxalobacteraceae, Porphyromonadaceae, Anaerotruncus, Desulfovibrio, Haemophilus and Holdemania were identified to be associated with endometriosis by the IVW method. The consistency of the effect estimates across all MR methods enhanced the robustness of the true association (Table 1). Specifically, the IVW estimate suggests that Porphyromonadaceae had a hazardous effect on endometriosis (OR = 1.27, 95%*CI*: 1.03–1.56, *P* = 0.027) (Table 1). Furthermore, the IVW estimate of Anaerotruncus indicated a suggestive hazardous effect against endometriosis (OR = 1.29, 95%*CI*: 1.07–1.55, *P* < 0.01) (Table 1). Additionally, the remaining five bacterial components Clostridiales_vadin_BB60_group (OR = 0.86, 95%*CI*: 0.78–0.95, *P* < 0.01), Oxalobacteraceae (OR = 0.91, 95%*CI*: 0.85–0.98, *P* = 0.014), Desulfovibrio (OR = 0.88, 95%*CI*: 0.78-1.00, *P* = 0.046), Haemophilus (OR = 0.89, 95%*CI*: 0.80–0.99, *P* = 0.039) and Holdemania (OR = 0.88, 95%*CI*: 0.78–0.98, *P* = 0.025) exhibited a negative causal direction with endometriosis, suggesting that they possess protective factors against endometriosis (Table 1; Fig. 3).

**Table 1:** Causal estimations of gut microbiota on endometriosis in the MR analysis

Bacterial taxa (exposure)		MR method	*F*-value	No. of SNP	OR (95%CI)	*P*-value
Family	Clostridiales_vadin_BB60_group	IVW	41.99	15	0.86 (0.78 ~ 0.95)	< 0.01
		MR-Egger		15	0.97 (0.74 ~ 1.27)	0.828
		Weighted median		15	0.86 (0.75 ~ 0.99)	0.038
		Weighted mode		15	0.87 (0.70 ~ 1.08)	0.216
		Simple mode		15	0.84 (0.66 ~ 1.06)	0.168
Family	Oxalobacteraceae	IVW	89.19	14	0.91 (0.85 ~ 0.98)	0.014
		MR-Egger		14	0.83 (0.63 ~ 1.10)	0.213
		Weighted median		14	0.95 (0.85 ~ 1.05)	0.304
		Weighted mode		14	0.99 (0.84 ~ 1.17)	0.904
		Simple mode		14	1.00 (0.83 ~ 1.19)	0.984
Family	Porphyromonadaceae	IVW	22.08	9	1.27 (1.03 ~ 1.56)	0.027
		MR-Egger		9	1.84 (0.71 ~ 4.80)	0.250
		Weighted median		9	1.06 (0.82 ~ 1.36)	0.677
		Weighted mode		9	1.03 (0.71 ~ 1.48)	0.888
		Simple mode		9	1.03 (0.66 ~ 1.61)	0.908
Genus	Anaerotruncus	IVW	26.63	13	1.29 (1.07 ~ 1.55)	< 0.01
		MR-Egger		13	1.13 (0.64 ~ 1.98)	0.681
		Weighted median		13	1.22 (1.00 ~ 1.49)	0.054
		Weighted mode		13	1.26 (0.96 ~ 1.66)	0.127
		Simple mode		13	1.26 (0.94 ~ 1.71)	0.152
Genus	Desulfovibrio	IVW	51.27	10	0.88 (0.78 ~ 1.00)	0.046
		MR-Egger		10	0.78 (0.54 ~ 1.11)	0.200
		Weighted median		10	0.92 (0.77 ~ 1.09)	0.318
		Weighted mode		10	0.92 (0.77 ~ 1.09)	0.732
		Simple mode		10	0.94 (0.69 ~ 1.28)	0.701
Genus	Haemophilus	IVW	60.90	9	0.89 (0.80 ~ 0.99)	0.039
		MR-Egger		9	0.90 (0.71 ~ 1.15)	0.432
		Weighted median		9	0.93 (0.81 ~ 1.07)	0.305
		Weighted mode		9	0.93 (0.78 ~ 1.10)	0.430
		Simple mode		9	0.93 (0.77 ~ 1.13)	0.497
Genus	Holdemania	IVW	50.31	14	0.88 (0.78 ~ 0.98)	0.025
		MR-Egger		14	0.78 (0.56 ~ 1.10)	0.184
		Weighted median		14	0.94 (0.81 ~ 1.09)	0.402
		Weighted mode		14	0.95 (0.77 ~ 1.17)	0.647
		Simple mode		14	0.95 (0.76 ~ 1.21)	0.705

MR, Mendelian randomization; SNP, single nucleotide polymorphism; OR, odds ratio; CI, confidence interval, IVW, inverse variance weighted

### Sensitivity analyses

**Fig. 4:**
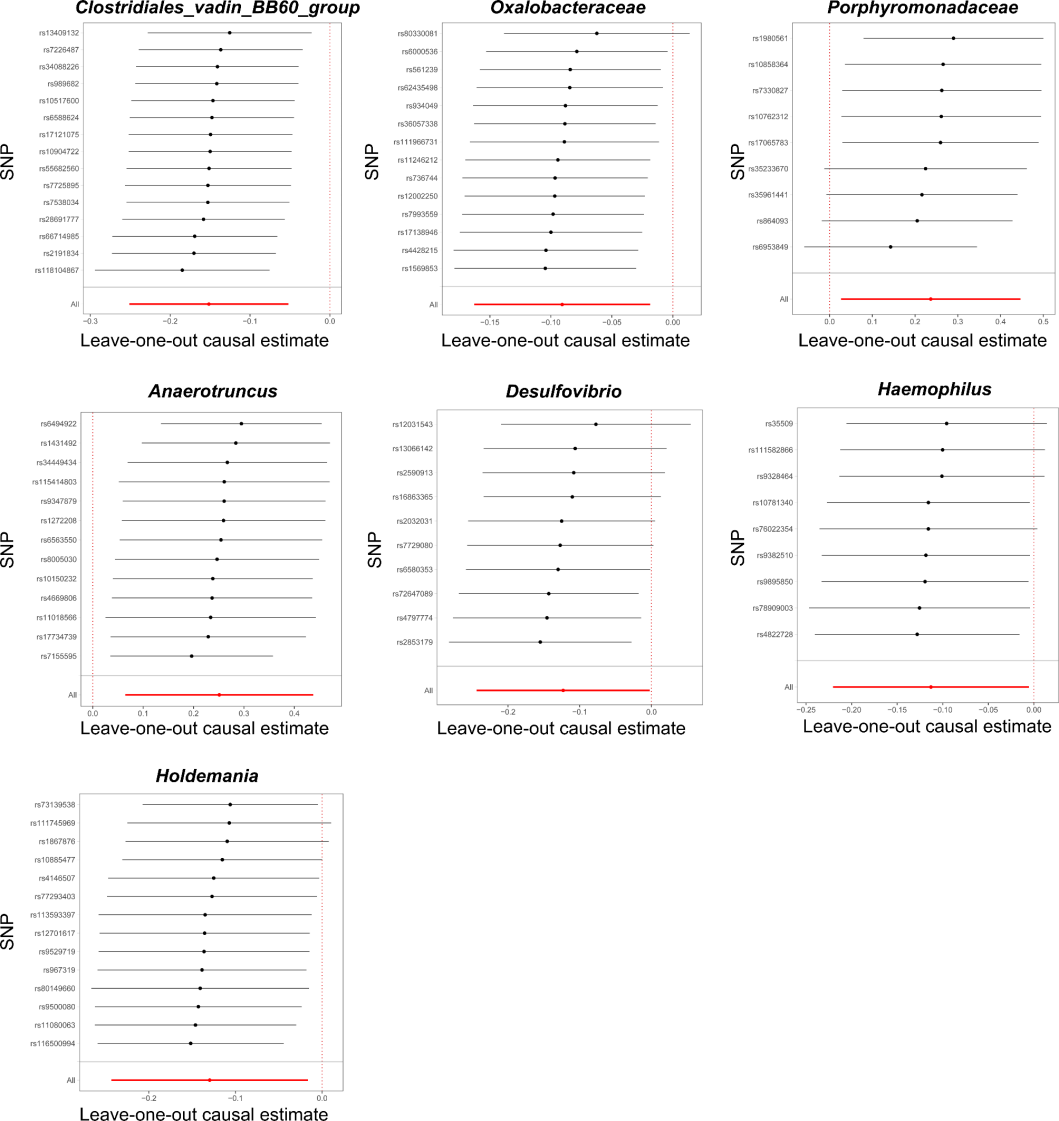
Leave-one-out plots for the causal association between gut microbiota and endometriosis. The sensitivity of the causal effect of different components of the gut microbiome on endometriosis was analyzed through leave-one-out analysis. The error bar represents the 95% confidence interval with the method of inverse-variance weighting. SNP, single nucleotide polymorphism

Of the seven causal associations examined, the F-statistics of the IVs ranged from 22.08 to 89.19, ensuring that weak IV bias was eliminated. MR analyses of Clostridiales_vadin_BB60_group, Oxalobacteraceae, Porphyromonadaceae, Anaerotruncus, Desulfovibrio, Haemophilus, and Holdemania with endometriosis exhibited no heterogeneity, as indicated by both Cochran’s Q statistic (IVW) and Rucker’s Q statistic (MR Egger), with *P*-values greater than 0.05 (Table 2). Moreover, the MR-Egger regression intercepts did not significantly deviate from null, suggesting that there was no evidence of horizontal pleiotropy for any of the associations (all intercepts with *P* > 0.05) (Table 2). Furthermore, leave-one-out sensitivity analysis revealed no single SNP driving the causal association signal (Fig. 4).

**Table 2:** Sensitivity analysis between gut microbiome and endometriosis

Bacterial taxa (exposure)	Heterogeneity				Horizontal pleiotropy		
	Rucker’s Q	*P*-value	Cochran’s Q	*P*-value	Egger-intercept	SE	*P*-value
	MR-Egger		IVW		MR-Egger		
Clostridiales_vadin_BB60_group	11.753	0.548	12.630	0.556	-0.012	0.013	0.366
Oxalobacteraceae	12.012	0.445	12.502	0.487	0.013	0.019	0.497
Porphyromonadaceae	9.425	0.224	10.265	0.247	-0.023	0.028	0.455
Anaerotruncus	21.327	0.030	21.776	0.040	0.009	0.019	0.497
Desulfovibrio	7.889	0.444	8.474	0.487	0.014	0.018	0.466
Haemophilus	4.058	0.773	4.067	0.851	-0.001	0.014	0.925
Holdemania	16.332	0.176	17.010	0.199	0.012	0.017	0.494

MR, Mendelian randomization; SE, standard error; IVW, inverse variance weighted

## Discussion

In the present study, we employed summary statistics of gut microbiota from the largest GWAS meta-analysis conducted by the MiBioGen consortium and the summary statistics of endometriosis from the FinnGen consortium R8 release data to perform two-sample MR analyses with the aim of establishing a genetic causal relationship between gut microbiota and endometriosis. Our findings revealed that increased genetic abundance of five gut microbiomes namely Clostridiales_vadin_BB60_group, Oxalobacteraceae, Desulfovibrio, Haemophilus, and Holdemania, was positively associated with a decreased risk of endometriosis. Furthermore, we identified that Porphyromonadaceae and Anaerotruncus may act as risk factors for endometriosis development. Importantly, our study is the first to utilize the MR concept to explore the causal relationship between the gut microbiome and endometriosis, offering fresh insights into future preventive and therapeutic approaches for this condition by targeting the specific dysbiosis of gut microbiota taxa.

The gut microbiome plays a crucial role in various physiological processes such as nutrient uptake, maintenance of the integrity of the gastrointestinal lining, regulation of the immune and endocrine systems, and protection against pathogenic insults [30, 31]. A range of observational and experimental studies have reported a correlation between the gut microbiota and endometriosis [7, 32–34]. This theory posits that eubiosis in the gut microbiome could regulate the homeostasis of circulating estrogen, while dysbiosis could disturb this equilibrium and contribute to estrogen-dependent conditions. Given that endometriosis is an estrogen-dominant condition, gut dysbiosis leading to abnormal levels of circulating estrogen could potentially contribute to the development of this disease [35]. Additionally, the secretion of β-glucuronidase and β-glucosidases by enteric bacteria could promote the deconjugation of estrogen, thereby increasing the reabsorption of free estrogens and resulting in higher circulating levels [36, 37]. Moreover, microbiota can be contributed to the development of endometriosis by promoting inflammation and hormonal dysregulation (through the estrobolome), altering cellular proliferation/apoptosis, metabolism, oxidative stress and angiogenesis [38, 39]. Therefore, the reproductive tract and intestines should not be considered as two completely independent systems, and their mutual influence should be considered in clinical practice.

The association between endometriosis and changes in the gut microbiome has been studied extensively. A recent study compared the gut microbiota of patients with endometriosis to that of healthy controls and observed alterations in both α and β diversities. The levels of Bacilli, Bacteroidia, Clostridia, Coriobacteriia, and Gammaproteobacteria were found to differ between the endometriosis and control group [40]. However, another study that collected rectal swabs at a depth of 3 cm found no difference in the gut microbiota between women with and without endometriosis [41]. The possible association between anogenital distance and endometriosis could also explain the transfer of organisms from the rectum to the vagina [42]. Nonetheless, previous investigations failed to establish causal relationships between endometriosis and the gut microbiome, and the sample sizes ranged from dozens to hundreds, lacking representative values for the overall population.

Our study identified Porphyromonadaceae and Anaerotruncatus as the risk factors for endometriosis. In previous studies, Porphyromonadaceae were positively correlate with aromatic amino acid metabolism, ammonia metabolism, and oxidative stress [43]. Similarly, Marina et al. observed significant concentrations of Anaerotruncatus in patients with endometrial cancer [44]. Furthermore, our study also confirmed that Clostridiales_vadin_BB60_Group, Oxalobactriae, Desulfovibrio, Haemophilus and Holdemania are protective factors against endometriosis. Arrones et al. identified Clostridiales_vadin_BB60_Group as a bacterial biomarker associated with T cell mediated autoimmune disease alopecia [45]. Chen et al. reported a positive correlation between the relative abundance of Desulfovibrio and beneficial genera including Oscillospira, Coprococcus and Bacteroides, while observing it to be negatively associated with harmful genera, such as Clostridium, Escherichia and Ralstonia [46]. Arthur et al. explored the changes in the gut microbiome among patients with metastatic melanoma and found that the gut microbiome was enriched with holdemania among ipilimumab plus nivolumab responders [47]. As far as our research is concerned, supplementing Clostriniales_ Vadin_ BB60_ Group, Oxalobactriae, Desulfovibrio, Haemophilus and Holdemania, and killing Porphylomonadaceae and Anaerotruncatus may become effective methods for preventing endometriosis. However, no study has explored the mechanism of action of these bacteria in endometriosis. Therefore, further randomized controlled trials are required to validate our findings.

This study has several advantages. First, the present study utilized data on the gut microbiome from 18,340 individuals across 24 cohorts and data on endometriosis from 114,119 European ancestry samples, rendering it more representative of the population. Second, MR analysis was employed to establish a causal relationship between the gut microbiota and endometriosis, thereby avoiding potential confounding variables and rendering the study more convincing than observational studies. Finally, the results of the sensitivity analysis indicated an absence of pleiotropy or heterogeneity, underscoring the statistical robustness of our findings.

It is crucial to acknowledge the limitations of this study. First, SNPs that met the genome-wide statistical significance threshold (5 × 10^− 8^) were too limited for further analysis. As a result, we included only those SNPs that met the locus-wide significance level (1 × 10^− 5^) in our study. Second, we were not able to conduct subgroup analyses because summary statistics, rather than raw data, were utilized. Third, we opted not to consider the results of multiple statistical corrections because of their excessively rigorous and conservative nature, which may overlook the potential causal relationship between endometriosis and certain gut microbiome taxa. This decision was based on biological plausibility. Fourth, due to the constraints of available data, this study is confined to patients diagnosed with ovarian endometriosis and thus unable to delve into other types of lesions, such as intestinal, peritoneal, and pelvic endometriosis.

## Conclusion

In conclusion, a comprehensive assessment was performed to examine the potential causal association between the gut microbiota and endometriosis. Our Mendelian randomization analysis identified two bacterial features with a positive causal direction towards endometriosis, while the other five bacterial features showed a negative causal direction. These causal associations could serve as valuable references for subsequent functional studies aimed at identifying the candidate microbial taxa. Moreover, our findings hold the potential to facilitate the development of novel strategies for the prevention and treatment of endometriosis via targeted interventions of specific gut microbiomes.

### Electronic supplementary material

Below is the link to the electronic supplementary material.

Supplementary Material 1

Supplementary Material 2

Supplementary Material 3

## Data Availability

The datasets analyzed during the current study are available in the MiBioGen repository, https://mibiogen.gcc.rug.nl/, and the FinnGen repository, https://r8.finngen.fi/.
