# Supplementary material for: Causal effects of gut microbiome on endometriosis: a two-sample mendelian randomization study
Source: BMC Womens Health. 2023 Nov 30;23:637. doi: 10.1186/s12905-023-02742-0 (PMC10687921; doi:10.1186/s12905-023-02742-0)
Supplement: Supplementary file 3 — Supplementary Material 3 [file 12905_2023_2742_MOESM3_ESM.docx]

**Supplementary legends**

**Table S1. All instrumental variables with significance.**

**Table S2. Instrumental variables used in MR analysis of the association between gut microbiota and endometriosis.**
